# Sex steroid hormone synthesis, metabolism, and the effects on the mammalian olfactory system

**DOI:** 10.1007/s00441-022-03707-9

**Published:** 2022-11-19

**Authors:** Tatjana Abaffy, Hsiu-Yi Lu, Hiroaki Matsunami

**Affiliations:** Molecular Genetics and Microbiology Department, Duke University Medical Center, Durham, NC 27710 USA

**Keywords:** Olfaction, Androgens, Estrogens, Steroidogenesis, Progesterone, Receptors

## Abstract

Sex steroid hormones influence olfactory-mediated social behaviors, and it is generally hypothesized that these effects result from circulating hormones and/or neurosteroids synthesized in the brain. However, it is unclear whether sex steroid hormones are synthesized in the olfactory epithelium or the olfactory bulb, and if they can modulate the activity of the olfactory sensory neurons. Here, we review important discoveries related to the metabolism of sex steroids in the mouse olfactory epithelium and olfactory bulb, along with potential areas of future research. We summarize current knowledge regarding the expression, neuroanatomical distribution, and biological activity of the steroidogenic enzymes, sex steroid receptors, and proteins that are important to the metabolism of these hormones and reflect on their potential to influence early olfactory processing. We also review evidence related to the effects of sex steroid hormones on the development and activity of olfactory sensory neurons. By better understanding how these hormones are metabolized and how they act both at the periphery and olfactory bulb level, we can better appreciate the complexity of the olfactory system and discover potential similarities and differences in early olfactory processing between sexes.

## Introduction

**Fig. 1:**
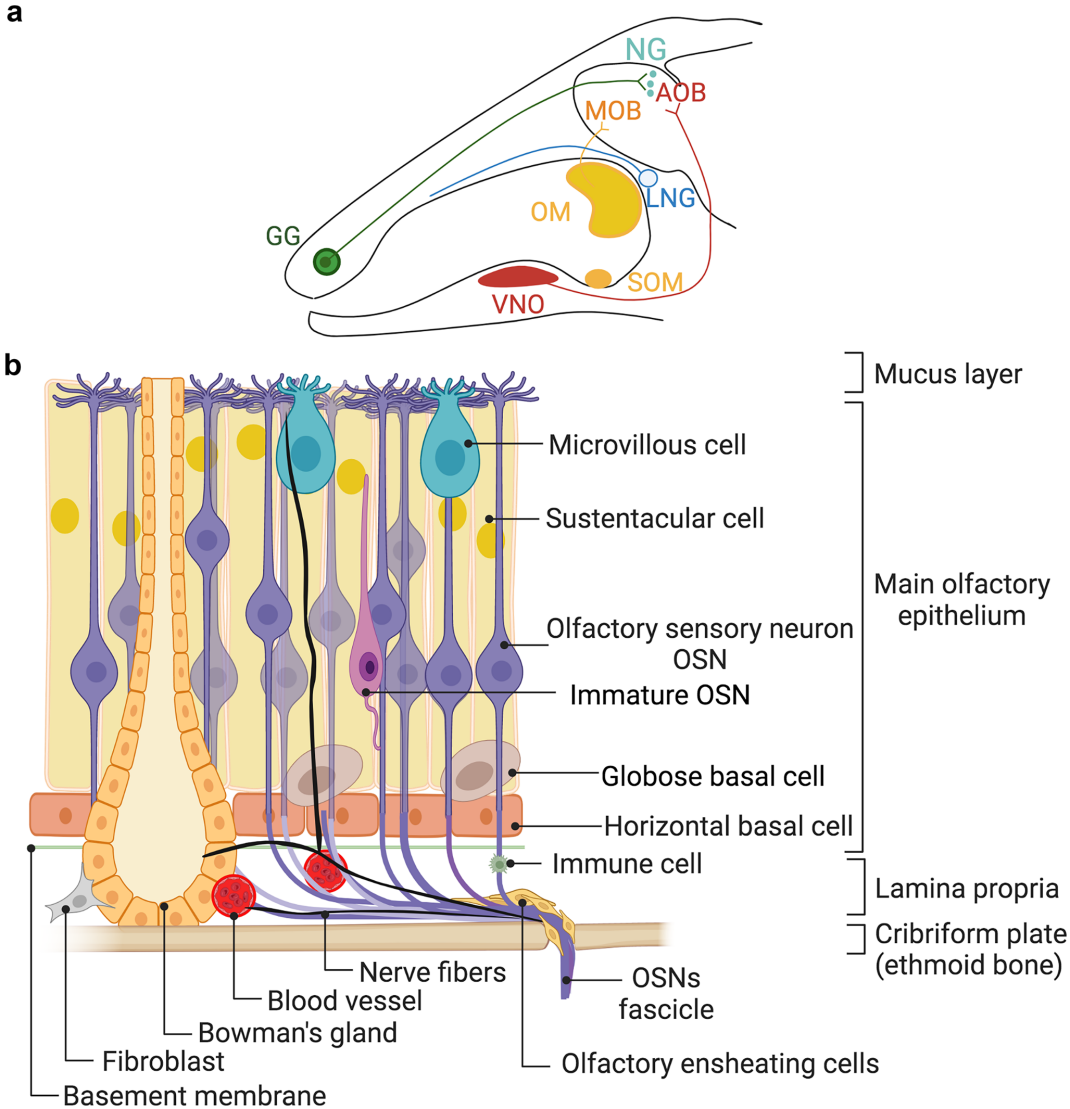
Olfactory system. **a** Cross-section of the mouse head with the chemosensory subsystems. Abbreviations: GG, Grueneberg ganglion; OM, olfactory mucosa; SOM, septal organ of Masera; VNO, vomeronasal organ; MOB, main olfactory bulb; AOB, accessory olfactory bulb; LNG, lateral nasal gland; NG, necklace glomeruli. **b** Schematic diagram of the olfactory mucosa (OM) with three layers, mucus layer, main olfactory epithelium, and lamina propria and their constituent cells. Main olfactory epithelium is comprised of OSNs, microvillar, and sustentacular cells and their precursor basal cells. Horizontal and globose basal cells are stem cells of the OE. For image clarity, immature sustentacular cells and two different types of microvillous cells were omitted. A basement membrane separates the olfactory epithelium from the lamina propria mainly consisting of Bowman glands, nerve fibers, olfactory ensheathing cells, blood vessels, and resident immune cells. Created with BioRender

The olfactory system is composed of the peripheral olfactory system and the olfactory bulb (OB). The peripheral olfactory system in mice consists of several anatomically segregated epithelia: the main olfactory epithelium (MOE), vomeronasal organ (VNO), Grueneberg ganglion (GG), and septal organ of Masera (Fig. 1a). While each of these areas is distinct, the function of some remains elusive (Fleischer et al. 2020). Odorant detection starts with olfactory receptor—odor interaction in the cilia of olfactory sensory neurons (OSNs). This interaction activates olfactory receptors, triggering cAMP transduction cascade, neuron depolarization, and generation of action potentials, which are transmitted to the main olfactory bulb (MOB). In addition to this canonical signaling pathway utilized by OSNs, other transduction pathways and projections exist. Activation of vomeronasal neurons cause transient receptor potential TRPC2 channels to open and signal transmissions to the accessory olfactory bulb (AOB) (Spehr et al. 2006). Meanwhile, activation of Grueneberg neurons (GG) generate cGMP, and the signal is transmitted to the necklace glomeruli (NG) in the OB (Fleischer 2021).

The main olfactory epithelium consists of heterogenous population of cells including OSNs and sustentacular, microvillar (MV), basal, and Bowman gland cells (Fig. 1b). Horizontal basal cells (HBCs), also known as reserve stem cells, are precursors of all cells in the olfactory epithelia (Fletcher et al. 2017). They differentiate to globose basal cells (GBCs) and immature olfactory sensory neurons before giving rise to mature OSNs. HBCs also give rise to microvillar and sustentacular cells (Fletcher et al. 2017). Various intrinsic, extrinsic, and paracrine signals affect OSN activity and thus odor sensitivity (Lucero 2013; Manzini et al. 2022; Bryche et al. 2021). These signals are either released from cells present in the olfactory epithelium and lamina propria, or they are derived from circulation. For example, OSNs release ATP and cannabinoids (Lucero 2013). Microvillar cells release acetylcholine, which can act as a paracrine signal to the OSNs, sustentacular cells (Ogura et al. 2011), and neuropeptide Y, which promote regeneration of OSNs following injury (Jia et al. 2013). Sustentacular cells and nasal gland cells release insulin, and insulin receptors are present in the OSNs, sustentacular, and basal cells (Lacroix et al. 2008). Insulin and leptin can decrease odor-induced OSNs activity, which can match smelling ability to satiety status (Savigner et al. 2009). Furthermore, insulin can also indirectly modulate olfactory signaling because it increases maturation and regeneration of OSNs following injury (Kuboki et al. 2021). Extrinsic innervation of the olfactory epithelium implicates potential modulation of olfactory responses by sympathetic and parasympathetic neurotransmitters such as norepinephrine and acetylcholine (Lucero 2013). In addition, trigeminal fibers are also present in the olfactory epithelium, which suggests potential modulation of olfactory signaling by substance P, calcitonin gene-related peptide (CGRP), and/or ATP (Lucero 2013).

The olfactory bulb (OB) is the first relay station of olfactory information (Brunert and Rothermel 2021). In addition to neurons, the OB contains other cell types including astrocytes, oligodendrocytes, and their precursors cells as well as vascular and immune cells (Nagayama et al. 2014; Lledo et al. 2008; Tepe et al. 2018; Brann et al. 2020). Due to this heterogeneity in the cellular composition of the olfactory bulb, the processing of olfactory information is subject to various neuromodulatory influences such as neuronal (GABA, glutamate) and non-neuronal factors/hormones (insulin, leptin, ghrelin) (Brunert and Rothermel 2021).

The role of sex steroid hormones in detecting and integrating socially relevant olfactory information has been established (Yang and Shah 2014; Wei et al. 2021; Liberles 2014). Sex steroid hormones modulate odor production, with male-specific odors being androgen-dependent. Similarly, odors of female mice depend on the estrous cycle. Estradiol and the sulfated metabolites of estrogens and progesterone are excreted in female urine and can activate both male and female vomeronasal sensory neurons (Alekseyenko et al. 2006; Nodari et al. 2008; Haga-Yamanaka et al. 2014). These effects are mediated by circulating sex hormones acting on the vomeronasal sensory neurons in the vomeronasal organ, but it is unknown whether they can act on the OSNs and if these hormones are also produced in the olfactory epithelium and the olfactory bulb. Sex steroid hormones are synthesized in the gonads, adrenal glands, placenta, and even in the nervous system (Baulieu and Robel 1990), which raises the question of whether the olfactory epithelium and olfactory bulb can also synthesize these hormones. In addition, can circulating androgens, estrogen, and progesterone modulate olfactory sensitivity? It is currently unknown if these hormones are transported from the capillaries, present in the lamina propria, into the olfactory epithelium, but the possibility exists. They may reach OSNs via sustentacular cells (Fig. 1b). For instance, transport of olfactotoxin methimazole from the blood to sustentacular cells has been previously described (Bergstrom et al. 2003).

The following major areas related to sex steroid hormones are the focus of this review:

1. Expression of steroidogenic enzymes in the OE and OB
2. The presence of sex steroid receptors in the OE and OB
3. Sex steroid hormones metabolism and transport within OE
4. Effects of sex and aging on the sex steroid hormone-related genes in the OE
5. Lateral nasal gland and Grueneberg ganglion
6. Effects of sex hormones on the morphology of the peripheral olfactory system and odorant-evoked signaling

We briefly discuss the effects of sex hormones on vomeronasal signaling but note that many reviews on this topic already exist (Jennings and de Lecea 2020; Petrulis 2013; Mohrhardt et al. 2018). Finally, we discuss important knowledge gaps that require further research.

### Expression of steroidogenic enzymes in the olfactory epithelium and olfactory bulb

**Fig. 2:**
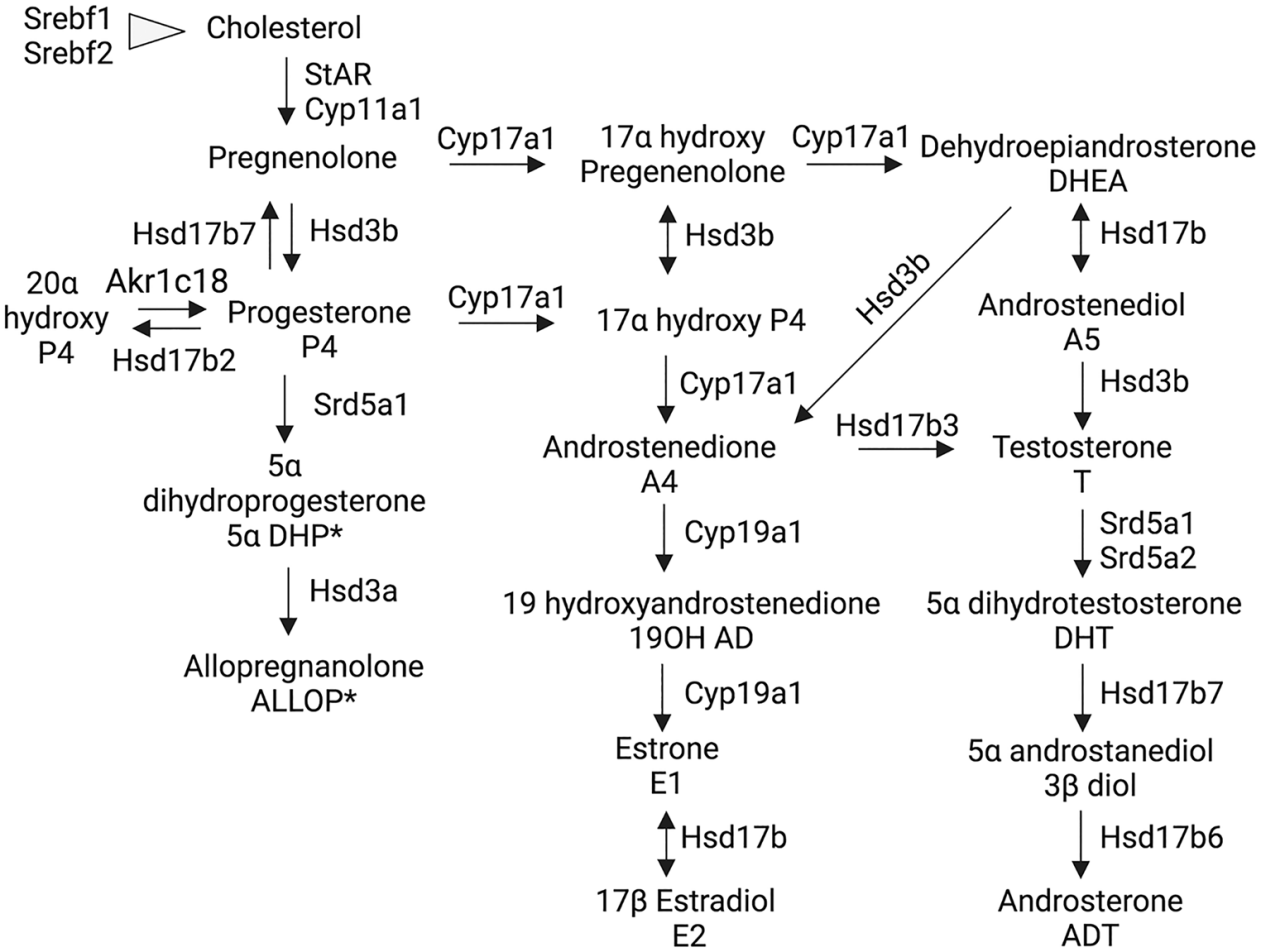
Steroidogenesis. Cytochrome P450 enzymes Cyp11a1, Cyp17a1, and Cyp19a1 reactions are indicated. Hsd3b reactions catalyzing synthesis of progesterone, androstenedione, and testosterone are also indicated. For clarity, specific Hsd17b isoforms in some reactions were omitted (discussed in more detail in Fig. 3). *Neuroactive steroids (ALLOP and 5aDHP) are defined as steroids able to alter neuronal excitability via interaction with GABA-A receptors. ALLOP* and 5aDHP* are positive allosteric mediators of GABA-A receptor and can influence sexual behavior

**Fig. 3:**
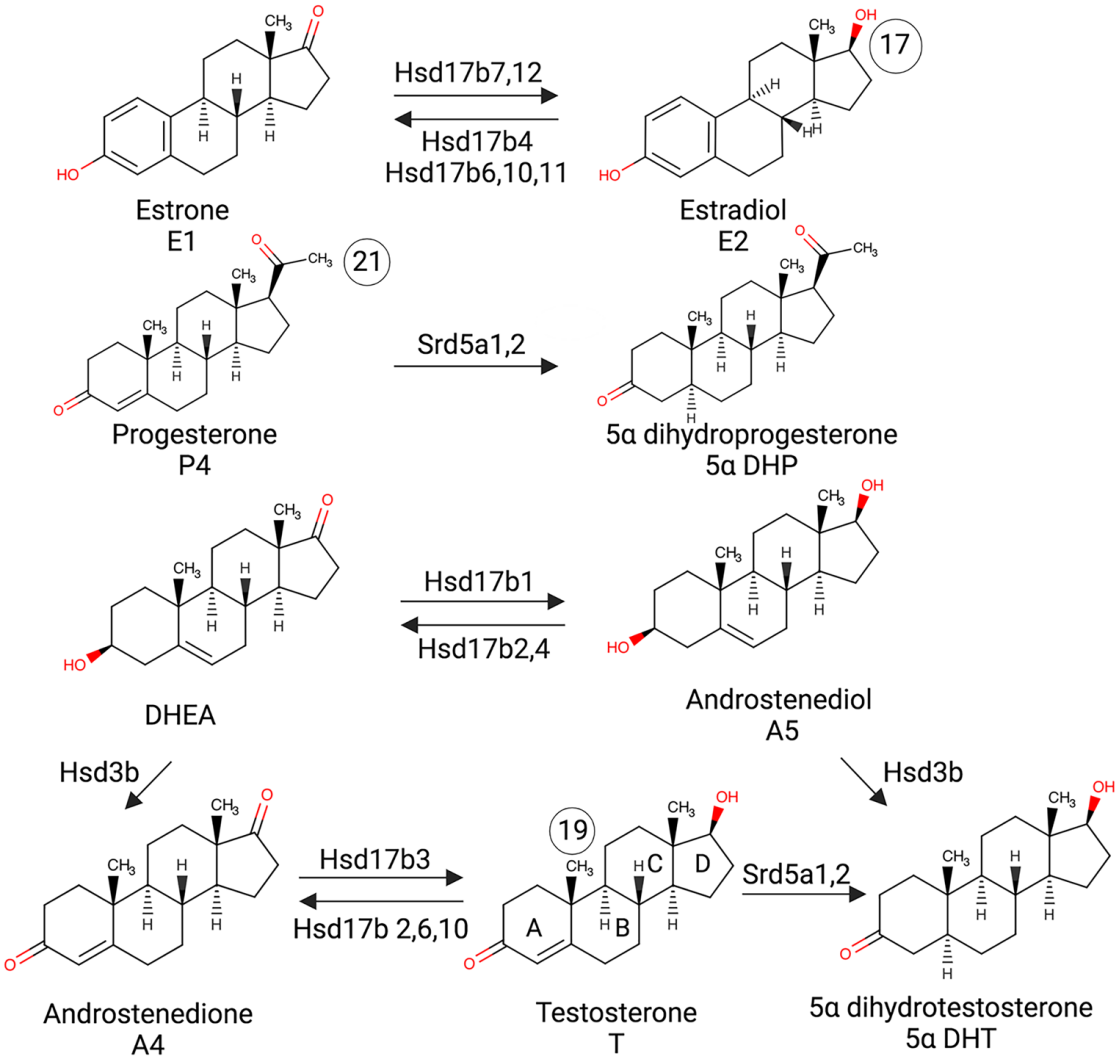
Steroidogenesis and the associated enzymes. Hsd17b acts on position 17 in the steroid structure (indicated in estradiol, C17). Different isoforms of Hsd17b catalyze different reactions. Hydroxy-forms are active and produce estradiol (E2), androstenediol (A5), and testosterone (T), while keto-forms are inactive (estrone [E1], DHEA, and androstenedione [A4]). Reduction of testosterone (C19, indicated) and progesterone (C21, indicated) by alpha-reductase (Srd5a1,2) to 5αDHT and 5αDHP is also presented. A, B, C, and D rings in the steroid structure are also indicated in the testosterone

**Fig. 4:**
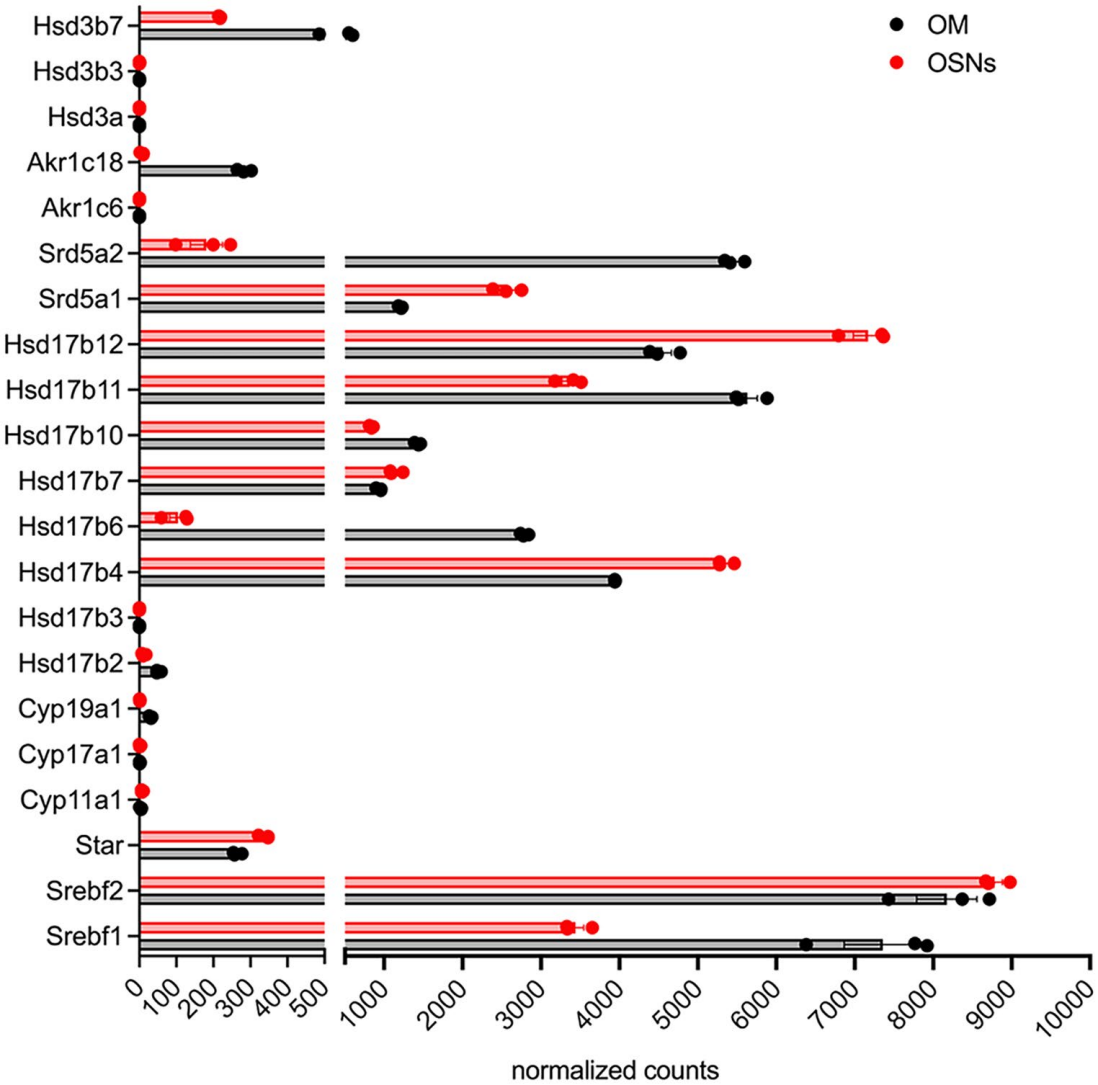
Normalized expression of genes involved in steroidogenesis in the OSNs and the olfactory mucosa (OM). OSNs were isolated from the heterozygous mice (2 males and 1 female) expressing GFP from the endogenous OMP promoter using a florescence activated cell sorting (FACS), while the OM samples were isolated from the separate matched control mice. The OM samples, in addition to the OSNs, contain sustentacular, basal, microvillous, and Bowman’s gland cells and also cells and fibers originating from the lamina propria, such as fibroblasts, resident immune cells, olfactory escheating cells (OECs), blood vessels, and nerve fibers (Fig. 1b). The gene expression level from the OM samples is *an average expression level* of each gene across these different cell types, thus potentially obscuring the heterogeneity in its expression. Raw sequencing counts were normalized for the whole transcriptome of the OM and about 10 million OSNs. Dataset from Saraiva et al. (2015).

**Fig. 5:**
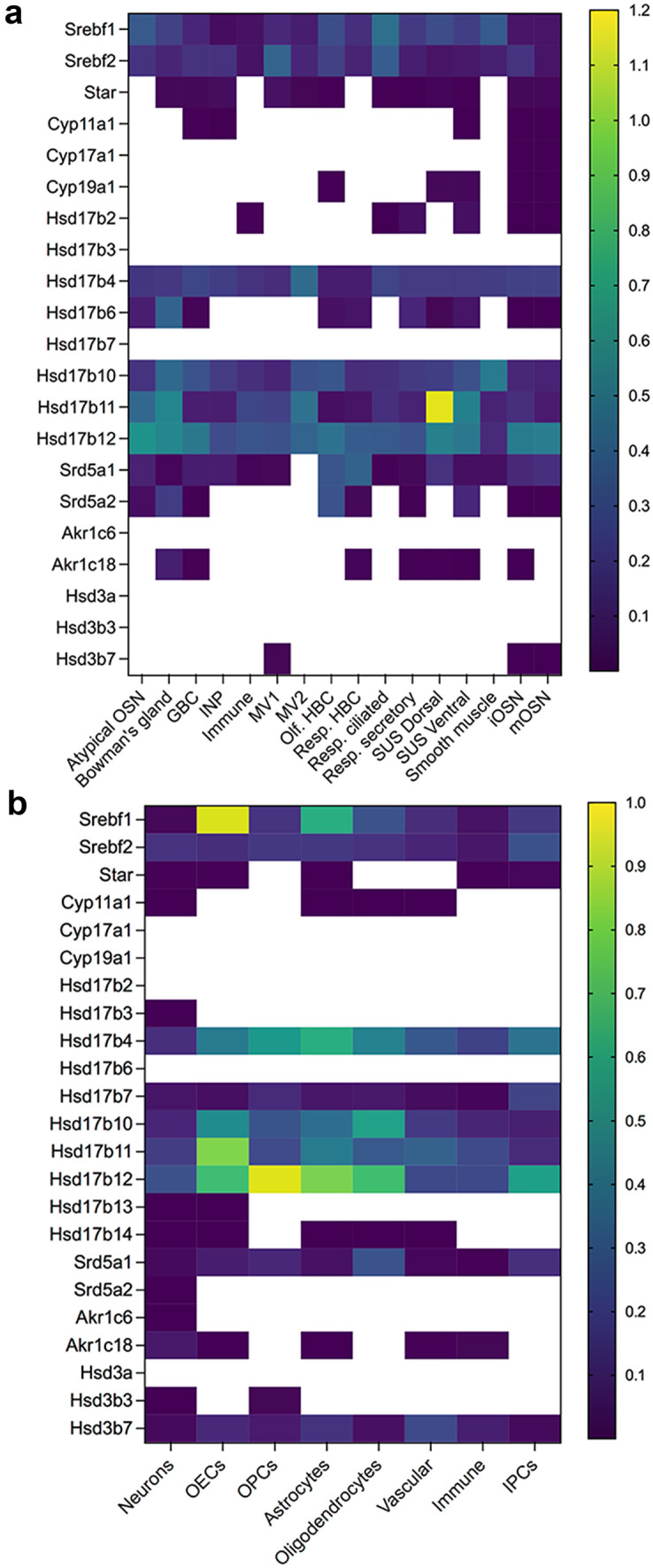
Heatmaps of genes encoding steroidogenic enzymes in the olfactory mucosa and olfactory bulb. **a** Heatmap of gene expression values across different cell types from the olfactory mucosa. Dataset from Brann et al. (2020). These values represent an average gene expression for a given cell type. Scale bar indicates logarithmic value of gene expression, with minimum set at 10^−7^. Empty cells indicate no-detectable levels. Microvillous cells MV1 are brush-like cells positive for Trpm5 + , while MV2 are ionocyte-like cells positive for Ip3r3. SUS sustentacular cells, INP immature neuronal precursors cells, iOSN immature OSNs, and mOSNs mature OSNs. **b** Heatmap of gene expression values across different cell types from the OB. Dataset from Brann et al. (2020). Empty cells indicate no-detectable levels. OEC olfactory ensheathing cells, OPCs oligodendrocytes precursors cells, and IPCs intermediate precursor cells

Synthesis of sex-steroid hormones is a complex process catalyzed by multiple enzymes (Figs. 2 and 3). Here, we first analyzed expression of steroidogenic enzymes from the transcriptomic data of OSNs and the olfactory mucosa (OM) published by Saraiva et al. (2015), (Fig. 4). Gene expression levels from these samples are *an average expression level* of each gene across different cell types present in the OE and lamina propria, thus possibly obscuring cell-specific levels due to the heterogeneity of cell types. In order to parse out which cells express which particular gene, we combined and reanalyzed single-cell transcriptomic datasets of the male mouse olfactory mucosa (GSE151346) and OB (GSE148360) that were previously published (Brann et al. 2020) (Figs. 5, 8, and 12). The cells from these datasets were selected based on the previously published markers from the single-cell RNA sequence analysis (scRNA – Seq) (Brann et al. 2020).

Here, we listed expression of steroidogenic enzymes from these two datasets, and also included results, when available, from the previous studies.

Cholesterol is a precursor of steroids, and its biosynthesis is regulated by the sterol regulatory element-binding transcription factor 1 (Srebf1) and 2 (Srebf2) genes. Both genes are found in the OSNs and OM, and their distribution remains constant across different cell types (Figs. 2, 3, and 4). In the OB, Srebf1 is highly expressed in olfactory ensheathing cells (OECs) and to a smaller extent in astrocytes (Fig. 5b). Steroidogenic acute regulatory protein (Star) mediates the transfer of cholesterol from the outer to the inner mitochondrial membrane (Clark et al. 1994; Stocco 2001). Low expression of Star is detected in the OSNs, several cells of the olfactory mucosa, and also in the OB (Figs. 2, 4, and 5). The presence of Star in the OB has been previously reported (Furukawa et al. 1998; Kim et al. 2002; King et al. 2002).

The first step in steroidogenesis is cleavage of the side chain of cholesterol by cytochrome Cyp11a1 (P450scc) (Fig. 2). Relatively low expression of this enzyme when compared to other genes was detected in the GBCs, ventral sustentacular cells, immature neuronal precursor cells (INP), iOSNs, and mOSNs (Fig. 5a).

The next important enzyme in steroidogenesis is cytochrome Cyp17a1. Cyp17a1 catalyzes hydroxylation of pregnenolone to 17α hydroxy pregnenolone and its subsequent conversion to dehydroepiandrosterone (DHEA), which is a precursor for the androgens androstenedione (A4), testosterone (T), and dihydrotestosterone (DHT). Very low levels of Cyp17a1 are present in the iOSNs and mOSNs, and no detectable expression was found in the OB (Fig. 5b).

17b hydroxysteroid dehydrogenase (Hsd17b) enzymes act on position 17 in the steroid backbone, where keto-forms are inactive and hydroxy forms are active and can access estrogen and androgen receptors (Fig. 3). Therefore, Hsd17b can modulate the biologic potency of sex steroid hormones (Mindnich et al. 2004). Hsd17b12 catalyzes the conversion of estrone to estradiol and is the most highly expressed member of Hsd17b in the OSNs (Fig. 4). Relatively constant expression of Hsd17b12 across all other cell types from the olfactory mucosa is observed (Fig. 5a). The strongest expression of Hsd17b12 in the OB was found in oligodendrocytes precursors cells (OPCs), followed by olfactory ensheating cells (OECs), astrocytes, and oligodendrocytes (Fig. 5b), indicating that conversion to active estradiol is important process in these OB cells. Hsd17b11 catalyzes the inactivation of estradiol and is expressed in both neuronal and non-neuronal cells, with expression highest in the sustentacular cells from a dorsal zone of the olfactory epithelium. Meanwhile in the OB, the expression is highest in the OECs. These results implicate a novel role for OECs in regulating and balancing estrogens in the close vicinity to the axons of the OSNs. The enzyme Hsd17b6 inactivates estrogens and androgens, and it catalyzes the conversion of estradiol to estrone, testosterone to androstenedione, 5a-androstanediol to (ADT), and ADT to epi-androsterone (epi-ADT) (Figs. 2 and 3). Hsd17b6 is abundantly expressed in non-neuronal cells, predominantly originating from Bowman gland cells. Hsd17b6 is absent from OB (Fig. 5b). Hsd17b4 catalyzes the inactivation of estradiol and conversion of androstenediol (A5) to DHEA and has slightly higher expression in OSNs than in non-neuronal cells (Fig. 4). Highest expression of Hsd17b4 is observed in microvillar MV2 cells, indicating their potential role in modulation of estrogen levels in the OE (Fig. 5a). Astrocytes, oligodendrocytes precursors cells (OPCs), oligodendrocytes, and OECs express higher levels of Hsd17b4 in the OB (Fig. 5b). Hsd17b10 catalyzes the inactivation of estradiol and testosterone, and although it is present in various cells of the olfactory mucosa, highest expression is observed in smooth muscle and Bowman gland cells (Figs. 4 and 5a). The highest expression of Hsd17B10 in the OB is present in oligodendrocytes and OECs (Fig. 5b). Hsd17b7 catalyzes the conversion of estrone to estradiol, progesterone to pregnenolone, and DHT to 5a androstanediol, and is present in OSNs and also in non-neuronal cells based on Saraiva data (Fig. 4) (Saraiva et al. 2015). Analysis of the single-cell transcriptomic dataset from Brann et al. (2020) did not show any detectable level of this enzyme in the olfactory mucosa samples (Fig. 5a); however, it is expressed in the OB samples across all cell types (Fig. 5b). Hsd17b3, which catalyzes the conversion of androstendione to testosterone, was not detected in the olfactory mucosa, but low levels are present in OB neurons (Figs. 4 and 5).

Aldo–keto reductase Akr1c18 converts and inactivates progesterone to 20a dihydroprogesterone and is expressed at low levels in sustentacular cells, Bowman’s gland cells, GBC, and immature OSNs (Figs. 4 and 5a).

Hydroxysteroid dehydrogenases 3a (Hsd3a) are also members of the aldo–keto reductase family of enzymes. Hsd3a (also known as Akr1c6) catalyzes transformation of 3-ketosteroids into 3-alpha hydroxysteroids and has an important role in the inactivation of androgens and also in the synthesis of neuroactive steroids such as ALLOP (Traish 2012; Robitaille and Langlois 2020). Hsd3a is not present in the whole olfactory mucosa or OB (Figs. 4 and 5). However, its abundant presence detected by in situ hybridization was previously reported in the periglomerular and mitral cells of the mouse OB, where it was colocalized with Srd5a1, suggesting active synthesis of ALLOP and 5a DHT (Guennoun et al. 1995; Agis-Balboa et al. 2006).

Hydroxysteroid dehydrogenases 3b (Hsd3b) catalyze the biosynthesis of testosterone, androstenedione, and progesterone (Fig. 2). Several isoforms of Hsd3b exist, but only Hsd3b7 is expressed in the immature and mature OSNs and microvillar MV1 cells (Figs. 4 and 5a). Currently, it is not clear if Hsd3b7 is also able to synthesize sex steroids, as information regarding this isoform 7 is limited to bile acid synthesis and not sex steroid synthesis (Shea et al. 2007). This enzyme is also present across all cell types in the OB (Fig. 5b).

5a reductase (Srd5a) enzymes catalyze the irreversible reduction of the double bond on the A ring at the 4,5 position in the testosterone (C19) and progesterone (C21) (Fig. 3) (Robitaille and Langlois 2020). Srd5a1 predominantly catalyzes the reduction of progesterone to 5a dihydroprogesterone (5a DHP) (Figs. 2 and 3), which can be further reduced by Hsd3a to allopregnanolone (ALLOP), a positive allosteric modulator of the GABA A receptor. However, given that Hsd3a is not detected in the olfactory mucosa or in the OB (Figs. 4 and 5), the neurosteroid ALLOP cannot be synthesized. However, 5a dihydroprogesterone produced by the Srd5a1 reaction can bind to the progesterone receptors (McEwen 1991; Dong et al. 2001) which are abundantly expressed in OSNs and in all non-neuronal cell types except MV2 cells, signifying a potential for progesterone signaling (Figs. 4 and 5a). Srd5a1 can also convert testosterone to dihydrotestosterone, and it is more expressed in HBCs, sustentacular cells of the dorsal zone of the olfactory epithelium, and OSNs (Figs. 4 and 5a). Srd5a2 enzyme is predominantly in Bowman’s gland cells and HBCs (Fig. 2c). In the OB, oligodendrocytes and intermediate precursor cells (IPCs) have slightly higher expression of Srd5a1, suggesting active androgen production (Fig. 5b). Intense Srd5a1 immunoreactivity was previously detected in the mitral and periglomerular cells of the OB (Agis-Balboa et al. 2006; Russell and Wilson 1994). Furthermore, constant expression of Srd5a1 in the OB at different ages and in both sexes has been reported (Kiyokage et al. 2005). In addition to Srd5a1, Srd5a2 immunolabeling in the OB has also been previously reported (Castelli et al. 2013).

Aromatase (Cyp19a1) converts androgens to estrogens (Abaffy and Matsunami 2021; Simpson et al. 2002). Its presence and activity in the brain was first reported by Naftolin and colleagues (Naftolin et al. 1971; Naftolin et al. 1975). By regulating the androgen/estrogen ratio, aromatase modulates various biological processes and contributes to sex-specific behavioral responses. Our current understanding of how aromatase-positive neurons are connected into brain networks was recently reviewed (Spool et al. 2021). Here, we summarize the current evidence to better understand whether and how aromatase, from the OE and OB, contribute to this network, possibly by modulation of olfactory sensitivity.

**Fig. 6:**
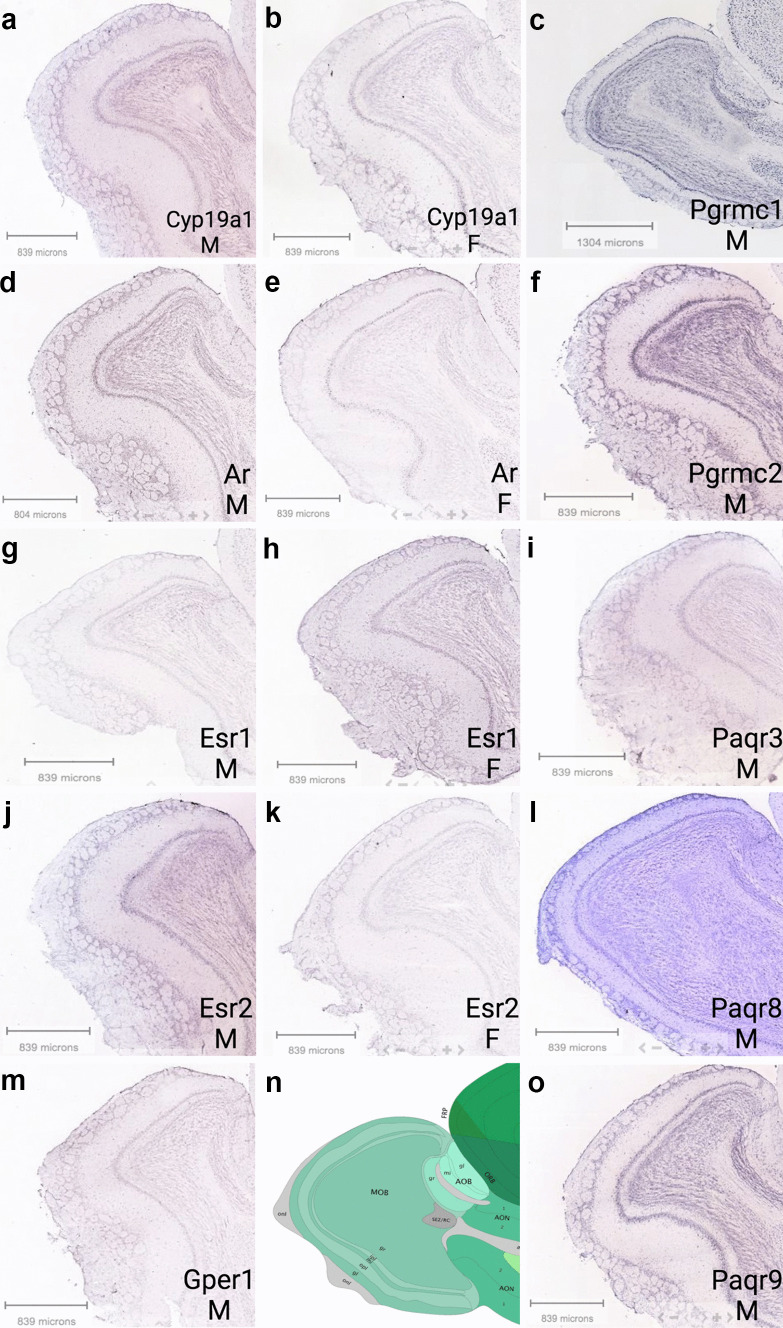
Localization of selected genes from the Allen Brain Atlas. Localization of aromatase (Cyp19a1, **a** and **b**), androgen (Ar, **d** and **e**), estrogen (Esr1, **g** and **h**; Esr2, **j** and **k**, and Gper1, **m**), and progesterone (Pgrmc1, Pgrmc2, Paqr9, Paqr3, and Paqr8, **c**, **f**, **i**, **l**, and **o**) receptors in the male (M) and female (F) mouse OB. Gper1 and progesterone receptor expression were only available from male samples. In situ hybridization of sagittal sections of the adult mouse OB, strain C57BL/6 J, mouse age 56 days. Scale bar is indicated in each panel. **n** Schematic of OB cell layers. Abbreviations: aco, fiber tracts; AON, anterior olfactory nucleus; FRP, frontal pore; gl, glomerular layer; gr, granule layer; ipl, inner plexiform layer; ml, mitral layer; onl, fiber tracts; opl, outer plexiform layer; ORB, orbital area; SEZ/RC, subependymal zone

Aromatase mRNA and enzymatic activity were detected in the *rat* OE through RT-PCR (Horie et al. 2017; Lupo et al. 1986). Several transcriptomics studies in *mice* have reported very low levels of aromatase, with FPMK < 1 (Saraiva et al. 2015; Kanageswaran et al. 2015; Ibarra-Soria et al. 2014). Brann et al. single-cell transcriptomic dataset revealed aromatase presence in immature and mature OSNs (Fig. 5a), but not in the OB (Fig. 5b) (Brann et al. 2020); however, a more sensitive method, such as in situ hybridizaton, revealed aromatase presence in different areas of the MOB and AOB. Strong staining was observed in the mitral, granular, and glomerular layers of the adult mouse OB, and it was much higher in males, as reported in the Allen Brain Atlas (http://mouse.brain-map.org/experiment/show?id=71381001) (Fig. 6). Aromatase expression and activity was also previously detected in the mitral cells of the developing *rat* MOB, as well as in the granule layer of the *rat* AOB (Horvath and Wikler 1999). Its presence was reported in *rat* necklace glomeruli and in the mitral layer of the rat MOB (Shinoda et al. 1989; Shinoda et al. 1990). More recently, strong aromatase expression was detected in the juxtaglomerular cells, and weak expression was detected in the mitral and granule cell layers in the *rat* OB (Hoyk et al. 2014). All aromatase-positive juxtaglomerular cells are also tyrosine hydroxylase/glutamic acid hydroxylase 67 (TH/GAD67)–positive cells, which are known to modulate interglomerular inhibition (Kiyokage et al. 2010; Zhou et al. 2020). Thus, products of the aromatase reaction released from these cells—mainly estradiol but also other steroids—may indirectly modulate olfactory processing by participating in interglomerular inhibition. This may be possible given that aromatase presence was previously detected in presynaptic terminals of zebra finch brain (Peterson et al. 2005), and emerging data demonstrate that sex steroids can act as neurotransmitters as reviewed by Rudolph and colleagues (2016). Future studies need to explore the potential physiological role of aromatase and in situ estrogen production and the potential modulation of olfactory signaling. Aromatase-positive neurons are also found in the anterior part of AOB. These neurons are sexually dimorphic and project to the medial amygdala (Billing et al. 2020). It is interesting to note that axons of these neurons express vomeronasal V1R receptors, which are activated by conspecific odors (mainly sulfated steroids) (Nodari et al. 2008; Isogai et al. 2011; Hammen et al. 2014). Abundant expression of aromatase is also present in the olfactory tubercle and bed nucleus of stria terminalis, further implicating aromatase involvement in the higher olfactory processing in mediation of social behavior (Stanic et al. 2014). This is, however, beyond the scope of this review.

### The presence of sex steroid receptors in the olfactory epithelium and olfactory bulb

#### Estrogen receptors

Estradiol classically signals via two receptors, Esr1 and Esr2. Along with the androgen receptor, they belong to the superfamily of nuclear steroid hormone receptors. These receptors are hormone-inducible transcription factors. Additionally, the membrane-bound G protein-coupled receptor Gper1, also known as Gpr30, was shown to mediate rapid or “non-genomic” effects of estrogen (Filardo et al. 2002). These effects are initiated at the cellular membrane and lead to activation of second messengers. Recently, however, it was discovered that Esr1 and Esr2 are also able to function via membrane-initiated effects (Srivastava et al. 2013).

**Fig. 7:**
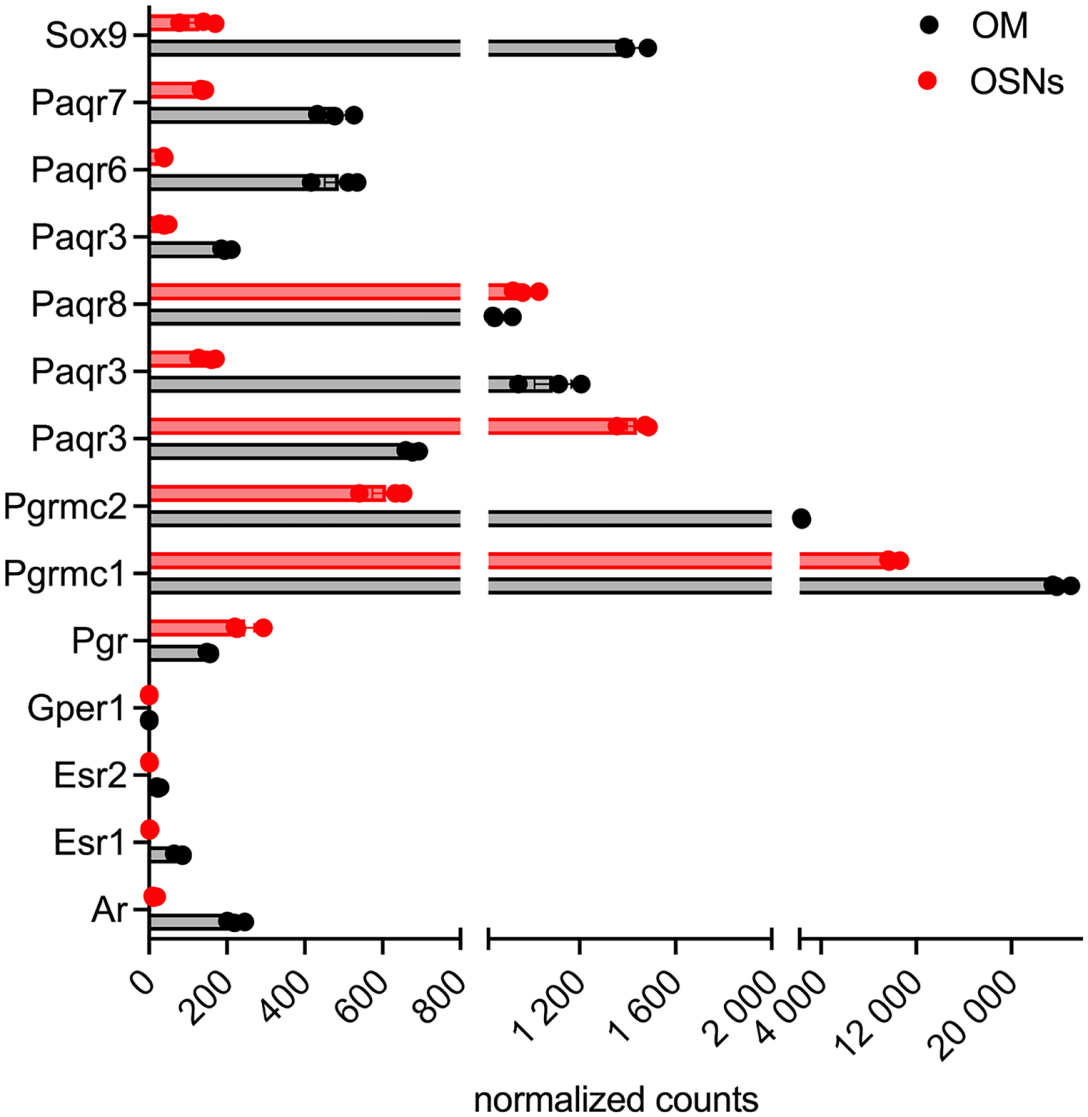
Expression of sex steroid hormone receptors and Sox9 transcription factor. Normalized expression of genes in the OSNs and the olfactory mucosa (OM), as described in Fig. 4. Dataset from Saraiva et al. (2015)

**Fig. 8:**
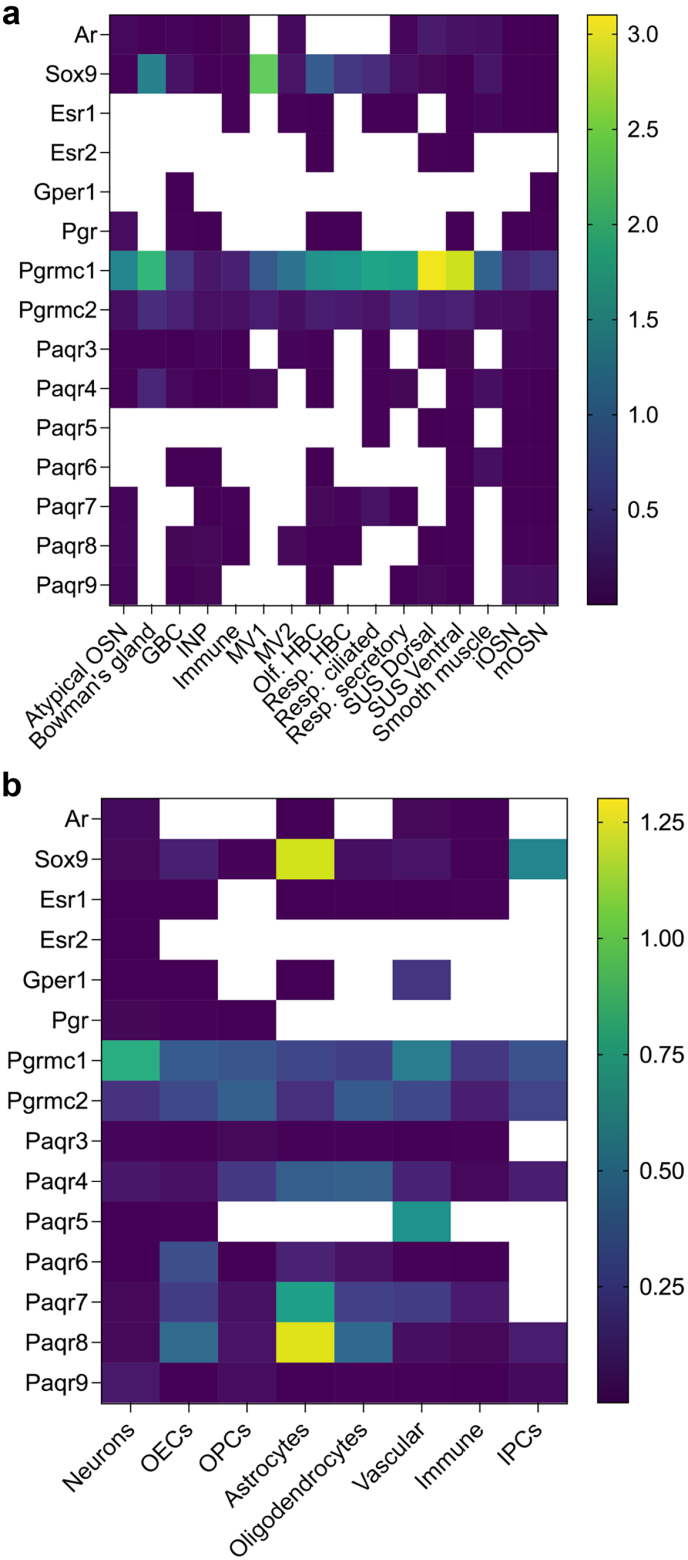
Heatmaps of sex steroid hormone receptors gene expression and Sox9 transcription factor values. **a** Across different cell types from the olfactory mucosa. Dataset from Brann et al. (2020). Empty cells indicate no-detectable levels. The highest expression of Pgrmc1 in the sustentacular cells is in agreement with data presented in Fig. 4. **b** Across different cell types from the OB. Results indicate the highest expression of Sox9 transcription factor and Paqr8 in the astrocytes, while Pgrmc1 is relatively abundant across all cell types in the OB. Dataset from Brann et al. (2020)

**Fig. 9:**
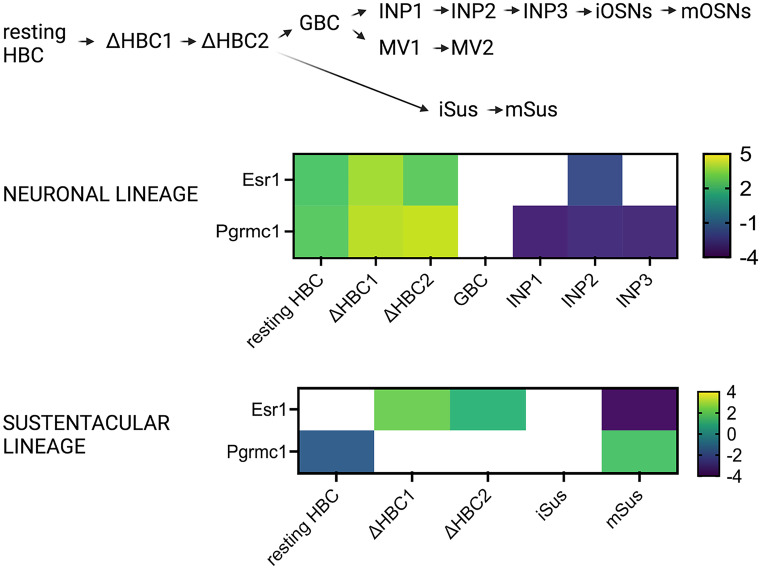
Lineage trajectory map of different cell clusters. Estrogen Esr1 and progesterone Pgrmc1 receptors show the highest expression in the HBCs of neuronal lineage, while Pgrmc1 has also high expression in the mature sustentacular cells (mSus). Dataset from Fletcher et al. (2017). Heat map display of log2 fold change in the Esr1 and Pgrmc1 gene expression (rows) between different cell clusters (columns) in the neuronal and sustentacular cell lineages

Esr1 is present in very low levels at the WOM (Fig. 7) (Saraiva et al. 2015), in immature and mature OSNs, ventral sustentacular cells, HBCs, MV2 cells, smooth muscle, and immune cells (Fig. 8a). Meanwhile, Esr2 is expressed in sustentacular and HBCs cells. Gper1 expression was detected in GBCs and mature OSNs (Fig. 8a). Immunolabeling of Esr1, Esr2, and Gper1 in the mouse OE has been previously reported (Pooley et al. 2015; Kanageswaran et al. 2016). A recent study from Fletcher et al. used a single-cell RNA-sequencing and lineage tracing analysis to identify developmental trajectories of neuronal and non-neuronal cells in the OE (Fig. 9) (Fletcher et al. 2017). Higher Esr1 expression was found in the HBCs compared to all other cell types of neuronal lineage, signifying a role of estrogen in the early trajectory of neuronal lineage. The same is true for the Esr1 in the sustentacular cell lineage. Furthermore, gene set enrichment analysis (GSEA) identified a “*hallmark estrogen response early*” as a significant signaling network that governs critical HBC transition to differentiation (Fletcher et al. 2017). These results demonstrate the importance of estrogen signaling in early trajectories of cell differentiation and development of OSNs and sustentacular cells (Fig. 9). In addition, it is interesting to note that Esr1 expression increases in more differentiated microvillar cell lineage and is higher in the MV1 than in the MV2 cells, data not shown (Fletcher et al. 2017).

In the OB, Esr1 expression is higher in females than in males, while the opposite is true for Esr2 (Fig. 6g and h). Changes in the expression of female Esr1 and Esr2 mRNA in the OB are observed during the estrous cycle (Reyes-Guerrero et al. 2010). Estrogens are directly involved in olfactory processing and the preservation of olfactory memories, which likely starts at the OB level (Sanchez-Andrade and Kendrick 2009; Dillon et al. 2013). Esr1 is expressed at low levels in almost all cell types of the OB, except IPC and OPC cells (Fig. 8b). Esr1 expression was also detected in female murine neurons of the ventricular-subventricular zone which produces new neurons in the postnatal brain (Isgor and Watson 2005). A subset of these neurons, which migrate along the rostral migratory stream into the OB, also express Gper1 receptor. Furthermore, astrocytes surrounding these migrating neuroblasts are aromatase positive, suggesting local estrogen production and potential involvement in the neuronal migration and olfactory neurogenesis (Haumann et al. 2020). These results indicate that estrogen has a role in olfactory neurogenesis in the OE and the OB.

#### Androgen receptor

Androgens signal via androgen receptor (Ar) which act as a transcription factor. Similar to Esr1 and Esr2, it can also induce rapid, non-genomic effects (Srivastava et al. 2013).

Androgen receptor is present in almost all cells of the OE, with a slightly higher expression in dorsal sustentacular cells (Figs. 7 and 8a); it is not present in HBCs or MV1 cells (Fig. 8a). These results are in agreement with the results from the Fletcher study (Fletcher et al. 2017), which did not identify androgen receptor as one of the most differentially expressed gene within the neuronal or non-neuronal cell lineage. However, GSEA of HBCs identified “*hallmark androgen response*” as an enriched and critical pathway for cell state transition. This may seem counterintuitive; however, a search for expression of other androgen-regulated genes revealed Sox9 gene as one of the differentially expressed transcription factors important for cell differentiation. Thus, androgen signaling via androgen-regulated gene Sox9 is critical for cell differentiation process in neuronal and sustentacular cell lineages. The highest expression of Sox9 gene was detected in non-neuronal cells, especially in MV1 cells, Bowman’s gland cells, and HBCs (Figs. 7 and 8a).

Androgen receptor expression in the OB has been reported in previous studies suggesting the possibility of androgen-receptor mediated signaling (Swift-Gallant et al. 2016; Simerly et al. 1990). Androgen receptor is necessary for male-typical olfactory preference, and it is conjectured that androgens act via non-neural androgen receptors to mediate this behavioral response (Raskin et al. 2009; Swift-Gallant et al. 2016). Analysis of single-cell transcriptomic dataset from Brann et al. revealed that in addition to the neurons in the OB, androgen receptor is also present in astrocytes, immune, and vascular cells, while Sox9 is also highly abundant in astrocytes and IPCs (Fig. 8b) (Brann et al. 2020). This suggests that non-neural androgen signaling in the modulation of male-typical olfactory preference in the OB is indeed possible.

#### Progesterone receptors

Progesterone is well characterized for its neuroprotective role in the CNS (Singh et al. 2013). Progesterone action is mediated by either classical nuclear or membrane receptors. The membrane-bound progesterone receptor Pgrmc1 is highly abundant in both OSNs and in the non-neuronal cells of the olfactory mucosa, and it is the most highly expressed sex steroid receptor gene in the OM and OSNs (Fig. 7) (Saraiva et al. 2015). Single-cell analysis reveals the highest expression in dorsal and ventral sustentacular cells and Bowman’s gland cells (Fig. 8a). Pgrmc1 is a multifunctional gene and in addition to being a progesterone receptor, it can a) act as a chaperone to shuttle cholesterol, heme, and various steroids (Cahill and Medlock 2017), b) bind to several cytochrome P450 enzymes and hence aide in xenobiotic metabolism (Hughes et al. 2007), and c) act as a carbon monoxide sensor (Kabe et al. 2016). What is the role of Pgrmc1 in the OE? Olfactory stem cell lineage analysis revealed high expression of Pgrmc1 in the sustentacular lineage group, with highest expression in the mature cells (Fig. 9) (Fletcher et al. 2017). This is opposite to Esr1 expression, as discussed above. These results allude the role of Pgrmc1 in shuffling estradiol into the cell for inactivation, as the expression of Hsd17b11 enzyme, which inactivates estradiol, is high in the sustentacular cells (Figs. 3 and 5a). The Fletcher study also reveals abundant expression of Pgrmc1 and Esr1 in the “resting HBC” when compared to all other cell types of neuronal lineage, which demonstrates the importance of progesterone and estrogen signaling in the differentiation of HBCs. Taken together, these results show progesterone’s role via Pgrmc1 in HBC differentiation and also in the function of mature sustentacular cells. Moreover, Pgrmc1 is detected in MV2 microvillous cells. Future research is warranted to reveal its function in these cells.

Paqr is a plasma membrane-bound progesterone receptor belonging to the progestin and adipoQ receptor family. Several members of Paqr family of receptors are present in the olfactory mucosa (Figs. 7 and 8a) (Saraiva et al. 2015; Kanageswaran et al. 2015, 2016; Brann et al. 2020). High abundance of these receptors in both neuronal and non-neuronal cells indicates active progesterone signaling. Furthermore, given that the levels of HSD3b1,2 and Cyp11a1 enzymes involved in progesterone synthesis are very low, the progesterone is presumably derived from the vascular system and not from local synthesis. Circulating progesterone increases during the diestrus phase and acts on Pgrmc1 receptors in the vomeronasal organ to silence the activation of female vomeronasal sensory neurons to some male urinary pheromones (Dey et al. 2015). Given the high abundance of Pgrmc1 in OSNs (Figs. 7 and 8a), it is possible that progesterone acts in a similar fashion to modulate olfactory responses in the OE. Indeed, in ex vivo experiments using submerged electro-olfactogram and whole-cell patch-clam recordings in the acute olfactory epithelium slices, progesterone, pregnenolone, and estradiol decreased odorant-evoked neuronal responses and odor-evoked cAMP levels. These effects were reversible (Kanageswaran et al. 2016). As immunodetection of Paqr8 and Paqr9 were more prominent than that of Pgrmc1, the authors concluded that Paqr receptors likely mediate these responses. In case of estradiol, these effects were mediated by Gper1 (Gpr30) estrogen receptor. Progesterone receptors are also present in the OB (Fig. 6c, f, i, l, and o), with Paqr8 and Paq7 showing the highest expression in astrocytes, while Pgrmc1 is highly expressed in neurons (Fig. 8b).

### Metabolic transformation and transport of sex steroids

#### Sulfation/desulfation

**Fig. 10:**
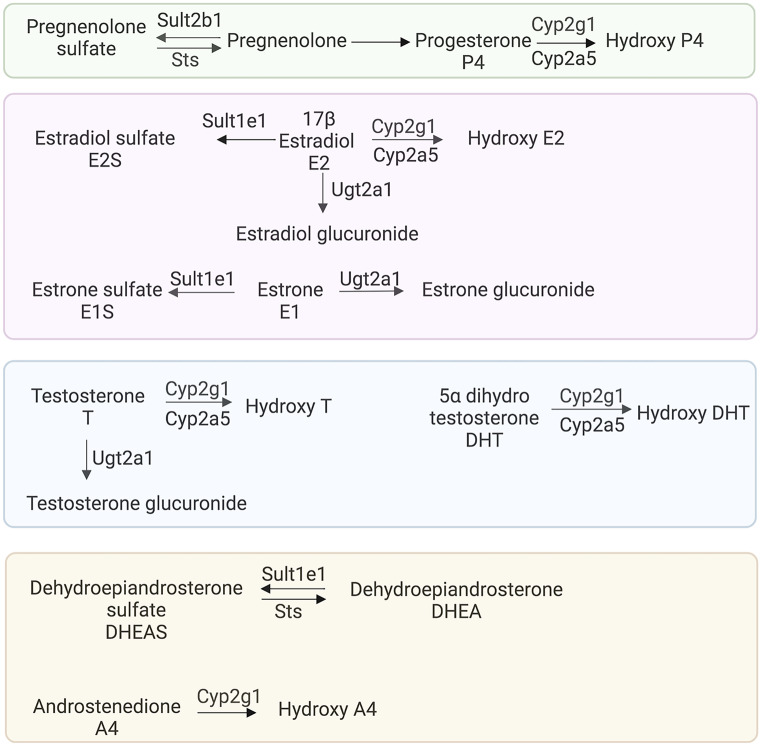
Schematic diagram of the enzymes involved in transport and metabolic transformation of sex steroid hormones

**Fig. 11:**
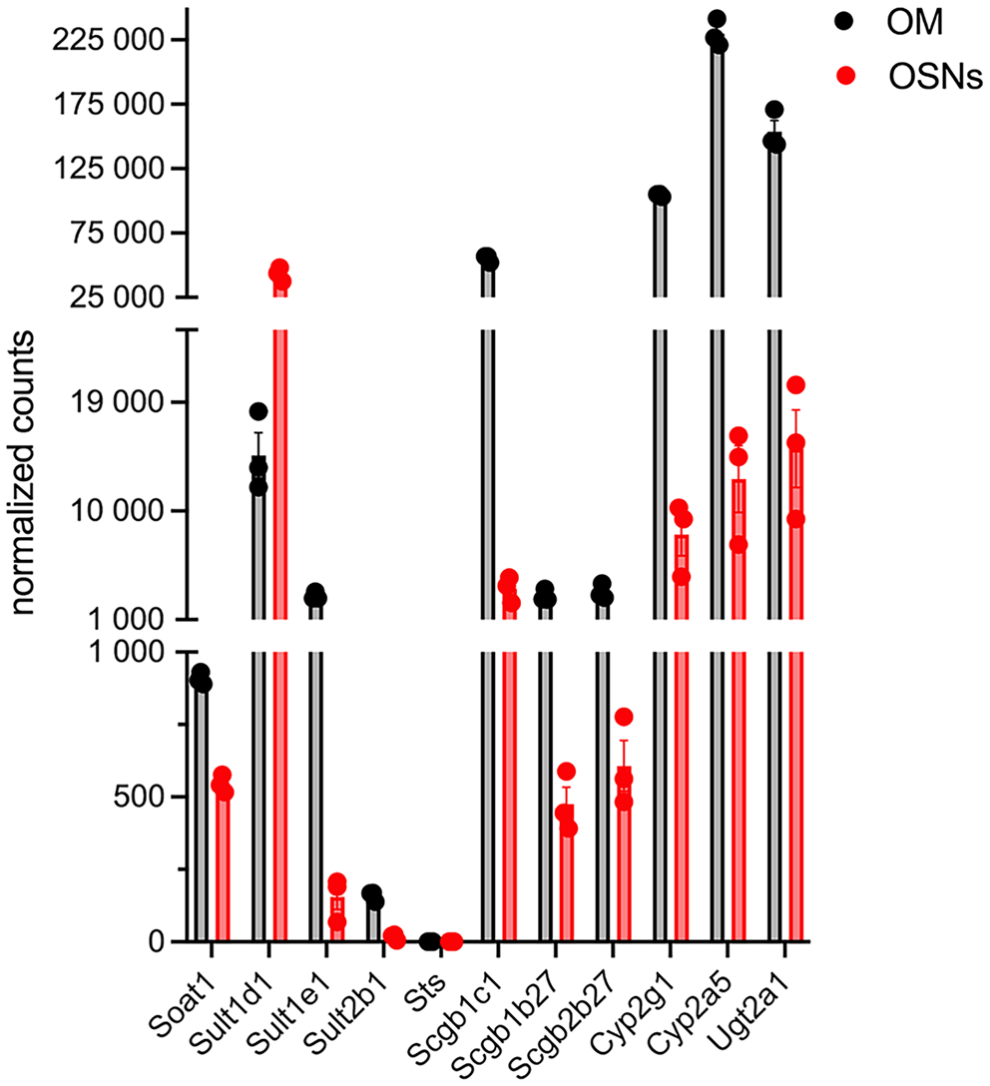
Normalized expression of genes involved in transport and metabolic transformation of sex steroid hormones in the OSNs and the olfactory mucosa (WOM), as described in Fig. 4. Dataset from Saraiva et al. (2015)

**Fig. 12:**
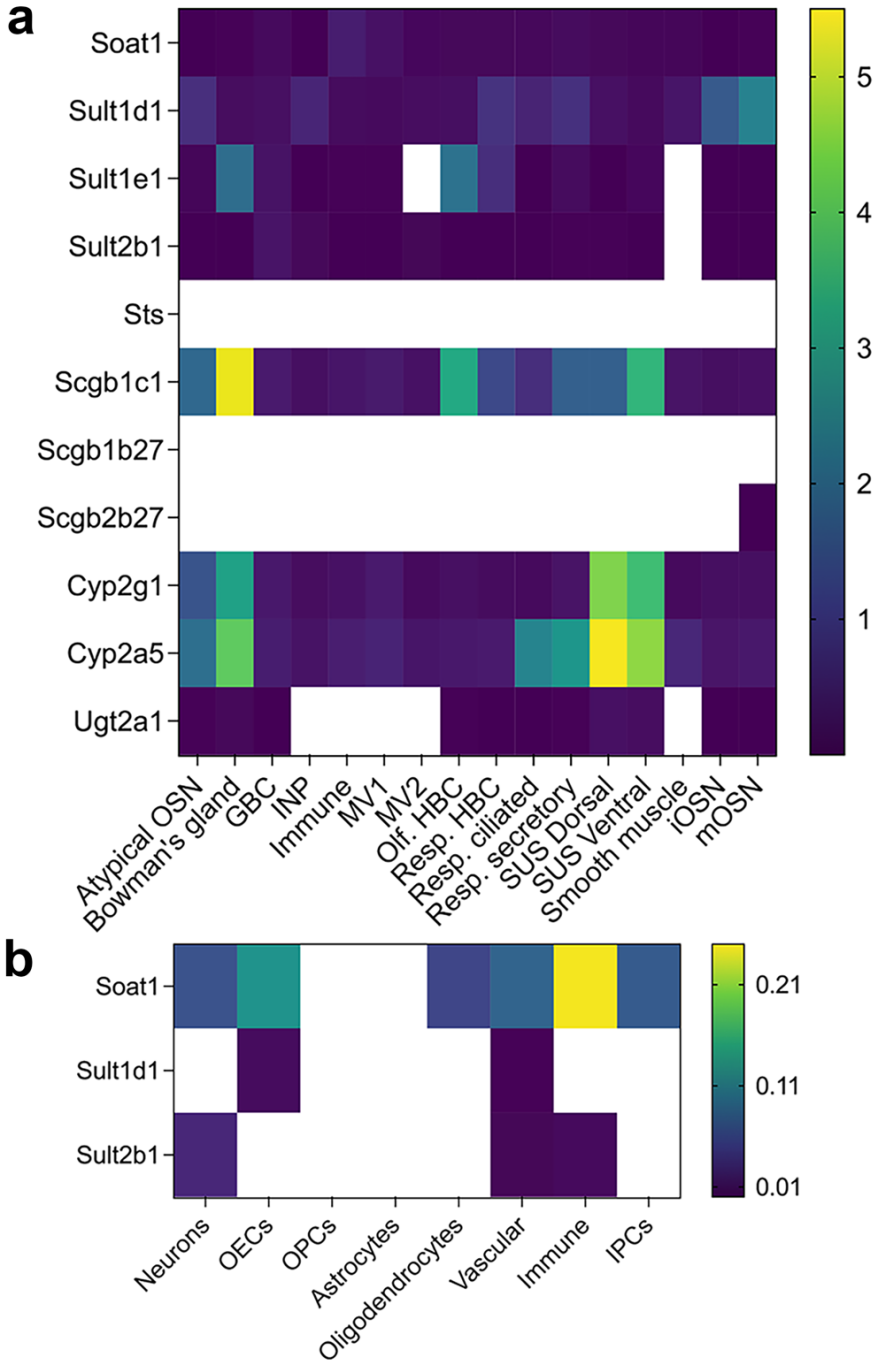
Heatmaps of genes involved in transport and metabolic transformation of sex steroid hormones. **a** Across different cell types from the olfactory mucosa. Dataset from Brann et al. (2020). Empty cells indicate no-detectable levels. **b** Heatmap of Soat1, Sulta1a, and Sult2b1 expression values across different cell types in the OB. Other enzymes and transport proteins listed in the OE have not been identified in OB. Dataset from Brann et al. (2020)

After steroids are synthesized, they are usually sulfated to increase hydrophilicity and expedite transport or excretion. The sulfation and desulfation process is controlled by sulfotransferases and sulfatases, respectively (Fig. 10) (Mueller et al. 2015). Sulfotransferases use 3’-phospho-5’-adenylyl sulfate (PAPS) as a sulfonate donor. Some sulfated steroids, such as DHEA-sulfate (DHEAS) and estrone sulfate (E1S), can act as circulating reservoirs of active hormones, and some can act as pheromones (Haga-Yamanaka et al. 2014). Sulfated steroids activate vomeronasal receptors and 4-pass transmembrane protein receptors (MS4A) (Greer et al. 2016). Sexual dimorphism in the response to various urine-derived sulfated steroids, such as epitestosterone sulfate, testosterone sulfate, and estradiol sulfate, exists and is modulated by sensory experience (Xu et al. 2016). The sulfotransferase Sult1e1 sulfonates estrogens and DHEA and is highly expressed in Bowman’s gland cells, and HBCs (Figs. 11 and 12a), revealing a tendency of these cells to sulfate estrogens and DHEA. Relatively low expression of Sult2b1, which sulfonates pregnanolone, was found in most non-neuronal cells of the olfactory mucosa (Figs. 11 and 12a). The sulfotransferase Sult1d1 is predominantly found in OSNs (Figs. 11 and 12a). Sult1d1 does not act on estrogens or DHEA, but rather on small phenols, dopamine, and 5-hydroxy tryptamine (Alnouti and Klaassen 2006; Tsoi et al. 2001; Sakakibara et al. 1998). An 11-fold difference in the expression of Sult1d1 was found in mature OSNs compared to immature OSNs (Tan et al. 2015). A > 100-fold difference in the expression of Sult1d1 was found in OSNs compared to other tissues such as brain, liver, muscle, and testes (Kanageswaran et al. 2015). When females are housed separately, female Sult1d1 expression after 43 weeks was higher than in sex-combined conditions (in separate conditions log2FC (F/M) = 0.297 with FDR 0.045, while in the sex-combined conditions, log2FC (F/M) =  − 0.071 with FDR 0.687). These results suggest that combined-sex housing reduces sex differences in the expression of Sult1d1 (Vihani et al. 2020). In the OB, Sult1d1 is expressed in OECs and vascular cells (Fig. 12b).

 Sulfated steroids can be transported across the plasma membrane via the solute carrier family of proteins. The sodium-dependent organic transporter 1/sterol O acyltransferase 1 Soat1 is more abundant in non-neuronal cells of the olfactory mucosa, though it is also present in OSNs (Figs. 11 and 12a) (Ibarra-Soria et al. 2014; Saraiva et al. 2015). In the OB, Soat1 highest expression is observed in the immune and OECs (Fig. 12b).

Steroid sulfatases (Sts) hydrolyze all steroid sulfates to their free steroid forms (Mortaud et al. 2010). The absence of these enzymes from the olfactory mucosa and OSNs, together with the presence of sulfotransferase Sult1e1 in the Bowman’s glands and HBCs, signify that steroid sulfation, and not desulfation, is a preferred pathway of non-neuronal cells in the olfactory mucosa. No sulfatases or sulfotransferases are detected in the OB.

#### Cytochrome Cyp2a5 and Cyp2g1 enzymes

The mammalian OE contains high levels of P450 monooxygenases involved in the metabolic transformation of potentially toxic chemicals, xenobiotics, sex steroids, and even some non-toxic odorants (Dahl et al. 1982; Ding 2003; Thiebaud et al. 2013). Cyp2a5 and Cyp2g1 are two major enzymes that are predominantly expressed in the sustentacular and Bowman’s glands cells (Figs. 10, 11, and 12a) (Ling et al. 2004; Thornton-Manning et al. 1997). Cyp2a5 is the most abundant gene in non-neuronal cells, with the highest expression in the sustentacular and Bowman’s gland cells (Figs. 11 and 12a). Cyp2a5 was recently used as a marker of sustentacular cells (Brann et al. 2020). Cyp2g1 is an olfactory-specific steroid hydroxylase that only acts on testosterone, estradiol, progesterone, androstenedione, and dihydrotestosterone, and not towards xenobiotic compounds (Hua et al. 1997; Gu et al. 1999). Cyp2g1 and Cyp2a5 are enriched > 100-fold in OSNs compared to other tissues (Kanageswaran et al. 2015). Both enzymes produce various hydroxylation products of sex steroids (Gu et al. 1999), which alludes that cells expressing these enzymes can metabolize sex steroids. All hydroxylated products of testosterone and estradiol have been identified, but the identities of two hydroxylated products of progesterone remain to be determined (Hua et al. 1997; Gu et al. 1999). Cyp2g1-null mice show decreased ability to metabolize testosterone and progesterone. They also have decreased expression of Cyp2a5 in the lateral nasal gland (Zhuo et al. 2004), suggesting that Cyp2g1 modulates the expression of Cyp2a5 and can indirectly increase metabolic transformation of androgens in the lateral nasal gland. So far, little is known about the regulation of olfactory Cyp2g1 (Ling et al. 2004). In humans, CYP2G1 is a pseudogene, as both copies of the gene have loss-of-function mutations (Sheng et al. 2000).

#### Ugt2a1 modulation of androgen availability

Ugt2a1, a major olfactory UDP-glucuronosyltransferase, catalyzes conjugation of UDP-glucuronic acid to an odorant molecule, which thus transformed cannot activate olfactory receptors ultimately leading to a termination of odorant signaling (Thiebaud et al. 2013). Ugt2a1 is a highly expressed gene and is present across most of cell types in the OE (Figs. 11 and 12a). This gene is enriched > 100 fold in OSNs compared to the other tissues (Kanageswaran et al. 2015). Strong immunostaining was present in the Bowman’s glands, sustentacular cells, and basal cells (Neiers et al. 2021). Recently, a genome-wide association study identified the association of the UGT2A1/UGT2A2 locus with COVID-19-related loss of smell (Shelton et al. 2022). Interestingly, Ugt2a1 can, in addition to odorants, catalyze the glucuronidation of androgens and 17-hydroxy estrogens (Sten et al. 2009; Jedlitschky et al. 1999; Itaaho et al. 2008). Since conjugated steroids cannot bind receptors, Ugt2a1 can therefore indirectly modulate the availability of androgens in the OE as 17-hydroxy estrogens are unable to bind estrogen receptors.

#### Steroid/sex hormone-binding globulin

Steroid/sex hormone-binding globulin (Shbg) binds androgens and estrogens with high affinity (Hammond 2016; Caldwell et al. 2006). Because bound steroids cannot enter cells, they are considered inactive. Thus, by binding sex steroids, Shbg can indirectly regulate sex steroid bioavailability. Relatively weak Shbg immunostaining was found in the subset of OSNs as well as in the olfactory mucosa (Ploss et al. 2014a, b). Similarly, low mRNA expression was detected in non-neuronal cells, and no expression of Shbg was detected in OSNs (Saraiva et al. 2015). In contrast, high expression was detected in the vomeronasal organ, but no sexual dimorphism was found (Ploss et al. 2014a, b). Shbg is expressed in the mitral cells of MOB and AOB (Caldwell et al. 2019).

### Effects of sex and aging on the sex steroid hormone-related genes in the olfactory epithelium

#### Early and subtle sex differences in the olfactory epithelium

**Fig. 13:**
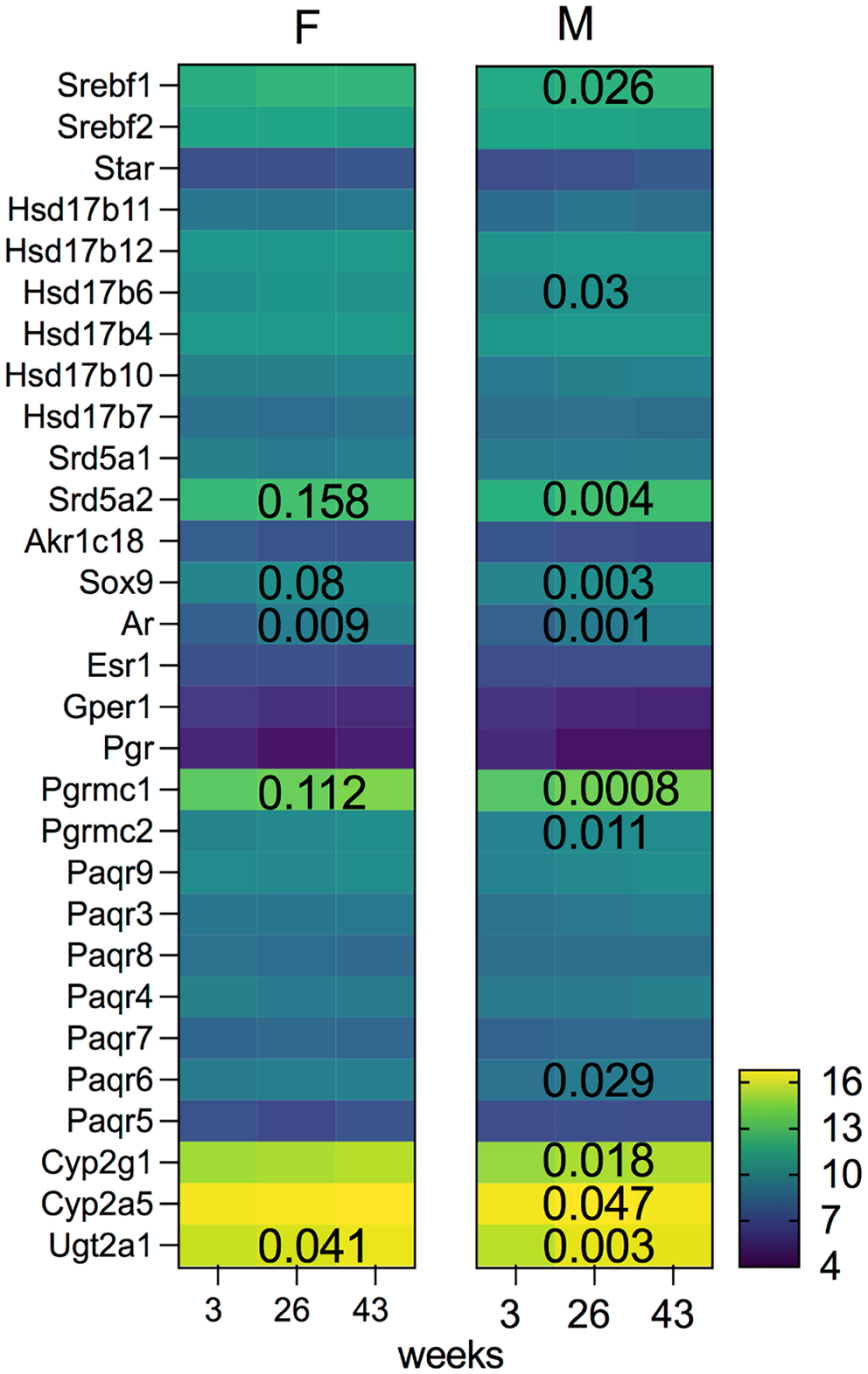
Effects of aging on the expression of sex steroid–related genes. Subtle sex differences in the expression of several genes were observed only at 3 weeks of age. The expression pattern of steroidogenic enzymes, receptors, and metabolic enzymes in female (F) and male (M) mice housed separately for 3, 26, and 43 weeks are shown. Mean expression data were log2 transformed. Significance level of changes in the expression of several sex steroid–related genes during aging is indicated with their *P*-values. Aging predominantly increases expression of sex steroid–related genes in male mice, whereas effects were less pronounced in female mice. A one-way ANOVA test was used to calculate *P-*values across these time points. Dataset from Vihani et al. (2020)

RNA-Seq analysis on the whole olfactory mucosa of male and female murine olfactory mucosa samples at 3, 26, and 43 weeks of age reveals a subtle difference between the sexes only at 3 weeks of age (Vihani et al. 2020). At 3 weeks of age, female mice have higher expression of the progesterone receptors Paqr9 and Paqr6 and higher levels of estradiol inactivation evidenced by increased expression of Hsd17b6 and Hsd17b11. They also have increased expression of the Srd5a2 and Srebf1 genes (FDR < 0.5 indicates differentially expressed genes, designated with an asterisk in Fig. 13). However, these differences were not present at 26 and 43 weeks of age. Similarly, a relatively high correlation in the transcriptomes of male and female olfactory epithelium was previously reported (Shiao et al. 2012).

#### Effects of aging

Changes in gene expression from 3 to 43 weeks were similar in most cases for both sexes. Ugt2a1 and androgen receptor expression increased in both sexes, and Srd5a2 gene expression increased in male mice (Fig. 13). Increased expression of androgen receptor and Srd5a2 prompted examination of the expression of Sox9, an androgen-regulated gene. Sox9 gene is a member of the sex-determining region Y type (SRY) high mobility group (HMG) box family of DNA binding proteins involved in a male sexual development. It also has an important role in maintaining neural stem cells in the embryonic and adult CNS (Scott et al. 2010) and during glial development (Kang et al. 2012). Male mice showed significantly increased expression of Sox9 at 26 weeks of age, during which they undergo puberty. These results suggest an increased role of androgens via Sox9 within the peripheral olfactory system as animals age and become sexually mature. Sox9 has been used as a marker of Bowman’s gland cells (Saraiva et al. 2019; Brann et al. 2020). Loss of Sox9 in the OB astrocytes results in decreased odor detection thresholds and discrimination, indicating that this androgen-regulated transcription factor is involved in olfactory signaling (Ung et al. 2021).

These results provide evidence of an indirect effect (not an androgen receptor-mediated process) of androgens on odor-induced olfactory signaling in the OB. This is supported by several behavioral studies in which male mice with genetically disabled olfactory signaling have a profound reduction in mating and intermale aggression (Mandiyan et al. 2005; Wang et al. 2006; Yoon et al. 2005).

Aging increases Pgrmc1 and Pgrmc2 expression similarly in both sexes; however, the results in females did not reach statistical significance, as greater variation in the expression was detected (Fig. 13). Paq6 receptor expression was significantly increased in male mice. These results suggest a potential role of progesterone in peripheral olfactory processing during puberty and adulthood and require further research.

### Lateral nasal gland and Grueneberg ganglion

#### Accumulation and secretion of androgens and secretoglobins from the lateral nasal gland

The mouse male lateral nasal gland (LNG), also known as Steno’s gland, is a secretory gland with a duct localized at the interior tip of the nose (Fig. 1a). Male mouse LNG has high levels of testosterone, while testosterone is undetectable in females (Zhou et al. 2009). These results indicate sexual dimorphism within the lateral nasal gland. Testosterone content is about fourfold higher in the LNG than in the olfactory mucosa. High testosterone content, the absence of aromatase, and very low transcript levels of 5-alpha reductase and testosterone-hydroxylase activity demonstrate that the LNG preferentially accumulates and secretes androgens, while very little androgen is metabolized within the gland itself (Zhou et al. 2009). In addition to testosterone, male LNG is abundant in androgen-binding or secretoglobin proteins encoded by Scgb genes, previously known as Abp genes. They bind and transport testosterone and dihydrotestosterone (DHT) (Karn 1998; Zhou et al. 2009). These results demonstrate that male LNG secretes testosterone and Scgb. The Scgb binds testosterone and is able to effectively transport it to the cells which can metabolize it to an even more potent androgen, DHT. Indeed, DHT and androstenedione (A4) were detected as main metabolic products of the radioactively labeled testosterone in the olfactory mucosa (Brittebo and Rafter 1984), suggesting importance of androgen signaling in the peripheral olfactory system.

Secretoglobins Scgb1c1, Scgb1b27, and Scgb2b27 were also found in non-neuronal cells of the olfactory mucosa, predominantly in Bowman’s gland cells, HBCs, and ventral sustentacular cells (Figs. 11 and 12a). Compared to other tissues (brain, liver, muscle, and testes), Scgb1c1 was > 100 × more enriched in the OE (Kanageswaran et al. 2015). When housed in sex-combined conditions, male mice had twice as much of Scgb1c1 than females did (Shiao et al. 2012). When housed separately, Scgb1b27 expression was significantly higher in males at 43 weeks of age (*P* = 2.85^−7^, FDR ≤ 0.05). Scbg2b27 was also differentially expressed, but the difference did not reach statistical significance (Vihani et al. 2020). When both sexes were housed together for a prolonged period of time, the difference was abolished. Thus, sex-combined housing reduces sexual dimorphism of the Scbg1b27 and Scbg2b27 genes. Differential expression of Scgb1b27 under sex-separated housing conditions has also been reported previously (van der Linden et al. 2018). These results suggest that increased expression of Scbg in sex-separated conditions implicates a necessity to bind testosterone. Indeed, a surge in testosterone has been observed when male mouse is exposed to the male conspecifics (male-male aggressive encounters) (Gleason et al. 2009).

#### Grueneberg ganglion neurons and aromatase positive necklace glomeruli

Grueneberg ganglion (GG) neurons are localized at the anterior end of the nasal cavity (Fig. 1a) (Grueneberg 1973; Fleischer 2021; Fuss et al. 2005). GG neurons mediate detection of alarm pheromones and predator odors, respond to cool temperatures, and can decode the threat of unfamiliar food (Brechbuhl et al. 2008; Chao et al. 2018; Brechbuhl et al. 2013, 2020; Bumbalo et al. 2017a, b). GG neurons project their axons to the necklace glomeruli (NG), which form a beaded ring of interconnected glomeruli localized on the border between the MOB and AOB (Fig. 1a) (Shinoda et al. 1989; Bumbalo et al. 2017a, b; Zimmerman and Munger 2021). Given that necklace glomeruli stain intensely for aromatase-associated placental antigen X-P2 (hPAX-P2), an understudied protein complex with aromatase (Shinoda et al. 1989; Shinoda et al. 1993), it is possible that pheromone signaling detected by guanylyl cyclase G, GC-G neurons is further modulated by aromatase in the necklace glomeruli. A unique population of OSNs also projects to necklace glomeruli; these neurons express receptor type guanylyl cyclase D (GC-D) (Juilfs et al. 1997), which detects diverse chemical stimuli (Leinders-Zufall et al. 2007). Although GC-G and GC-D axons project to different necklace glomeruli (Matsuo et al. 2012), it is unclear which glomeruli—GC-G or GC-D—correspond to the aromatase-associated placental antigen X-P2 positive glomeruli reported by Shinoda (Shinoda et al. 1989). Further research is necessary to identify aromatase-positive cells in the necklace glomeruli and to delineate their role in chemosensation.

### Effects of sex hormones on the morphology of the peripheral olfactory system and odorant-evoked signaling

Sex hormones act throughout the entire brain to directly or indirectly affect gene expression, regulate a variety of signaling pathways, and induce sex-dependent differences. These differences arise from locally synthesized and/or circulating sex steroids, different levels of sex steroids during the critical perinatal phase of the brain development (so-called organizational effects), and differences in sex chromosome effects (McEwen and Milner 2017). The inherent inequality of X and Y genetic material in the two sexes has effects throughout the body, not just on the gonads (Arnold 2017). In particular, the Y chromosome encodes several genes that eventually make males different from females, e.g., Sry gene, while the number of X chromosomes affects responses to various diseases and also behavior (Arnold et al. 2016).

Hormonal status can influence the odor detection threshold and odor discrimination, and here we review existing research related to the sex-steroid effects and differences in the morphology and function of the rodent peripheral olfactory system.

#### Effects of circulating estrogens on OSNs

Circulating estrogens positively affect OSN development, morphology, and synaptogenesis. The effects of circulating estrogens on the olfactory system were predominantly studied in ovariectomized mice. Early studies documented the protective role of estrogen against 3-methylindole-induced olfactory loss (Dhong et al. 1999). Estradiol treatment increased the thickness of the olfactory epithelium, induced proliferation of basal cells, increased the number of mature neurons, increased synaptophysin immunostaining in the OB glomeruli, and increased synapse formation between new OSNs and OB neurons (Nathan et al. 2012, 2010). Together, these results show that estrogens affect neuronal development, epithelial morphology, and synaptogenesis in the peripheral olfactory system. As discussed in “The presence of sex steroid receptors in the olfactory epithelium and olfactory bulb” section, estrogen signaling is critical for development of OSNs and sustentacular cells. Previously, it has been reported that estrogen acts via Esr1 receptors to increase neurite growth in OSNs (Pooley et al. 2015).

#### Estrogen effects on glomerular activity and odor discrimination

Using a habituation/dishabituation test to detect and investigate volatile urinary odors from conspecifics, it was observed that female mice have a lower detection threshold than male mice do (Baum and Keverne 2002). In contrast, Wesson et al. observed enhanced urinary odor discrimination in female aromatase knockout (ArKO) mice (Wesson et al. 2006), which have lower estrogen levels. These results are inconsistent. However, the type of behavioral assay used (food-motivated odor discrimination vs habituation/dishabituation) and the complexity of hormonal changes induced by ArKO (e.g., ArKO mice have upregulated Esr1 receptors (Agarwal et al. 2000)) complicate interpretation of the results.

Ex vivo patch-clamp recordings from the OSNs revealed that estradiol and progesterone decrease odorant-evoked signaling, as discussed earlier (Kanageswaran et al. 2016). However, results in vivo demonstrated much larger spatiotemporal pattern of glomerular activity in female than in male mice (Kass et al. 2017). Gonadectomy (GDX) in females, which lowers levels of circulating estradiol, also decreased but did not completely eliminate glomerular activation upon odor presentation. These results suggest that (a) circulating estradiol may increase odorant-evoked signaling in females, thus potentially leading to increased olfactory ability, and (b) gonadal hormones contribute to the modulation of olfactory circuitry in the olfactory bulb.

#### Effects of androgens and aromatase on the olfactory and vomeronasal system and signaling

Similar to estrogens, the effects of androgens on the olfactory system were studied first in castrated animals. Castration changed the morphology of the OE, which was counteracted by testosterone treatment (Balboni 1967). Castration also decreased odor detection (Doty and Ferguson-Segall 1989). Furthermore, it was demonstrated that testosterone treatment of GDX male mice was required for response to female pheromones and activation of the accessory olfactory pathway (Paredes et al. 1998). These results indicate that male preference for female olfactory cues is an androgen-dependent process. More recently, Schellino and colleagues demonstrated a critical role of circulating testosterone in the modulation of pheromone-induced adult neurogenesis in the AOB (Schellino et al. 2016). GDX in males increased glomerular recruitment upon odor presentation, implicating that androgens may suppress olfactory signaling (Kass et al. 2017). These results are in contrast to the effects of estrogen on the female OB and reveal sexually dimorphic activation of glomeruli by sex hormones.

Androgens and aromatase are critical for vomeronasal organ and AOB development during the perinatal period. Strong aromatase immunoreactivity was detected in the developing male and female vomeronasal organ and AOB (Horvath and Wikler 1999), and castration at postnatal day 1 had a profound, but reversible, effect on the postnatal development of vomeronasal organ (Segovia and Guillamon 1982). Application of testosterone during the early postnatal period increased the number of mitral cells in females, while orchiectomy in males decreased their number, suggesting perinatal hormonal regulation of AOB development (Valencia et al. 1986). These results are indicative of androgen and aromatase/estrogen involvement in the development and maturation of the vomeronasal organ and AOB.

Aromatase is abundantly expressed in medial amygdala neurons, which have been implicated in regulation of territorial aggression, parenting, social investigation, and other sex-specific social behaviors elicited by chemosensory cues. AOB neurons project to aromatase-positive neurons in the medial amygdala, and these projections are sexually dimorphic (Billing et al. 2020). However, the adult AOB also contains aromatase-positive neurons that originate from the ventral portion of the medial amygdala and are localized in the granule cell layer of AOB (Inbar et al. 2022). These results suggest that aromatase activity and consequent modulation of the androgen-to-estrogen ratio within the AOB are involved in the regulation of sensory representations at the olfactory bulb level, and therefore, they may give rise to sex differences.

## Conclusions and future perspectives

In summary, this review reveals several important facts. Low expression of enzymes involved in steroidogenesis in the OSNs (Cyp11a1, Cyp17a1, and Cyp19a1) suggests limited de novo steroidogenesis of sex steroid hormones. The presence of both activating and inactivating enzymes of estradiol indicate a tight control and balance of estrogen levels in the OSNs.

High expression of estrogen and progesterone receptors, Esr1 and Pgrmc1, in the HBCs of neuronal lineage suggests active role of estrogen and progesterone in the cellular differentiation and development of OSNs. Furthermore, results also indicate active androgen signaling via androgen-regulated gene Sox9, in the neuronal and sustentacular cell lineages.

Based on the profile of 17b hydroxysteroid dehydrogenases, dorsal sustentacular cells have a strong tendency to inactivate estradiol (high expression of Hsd17b11), while high abundance of progesterone receptor Pgrmc1 in these cells suggests active progesterone signaling and possibly even modulation of early olfactory processing. Furthermore, high expression of Sox9 in the microvillar MV1 cells indicates potential for androgen signaling.

Non-neuronal cells of the olfactory bulb also exert a tight control of androgens and estrogens. Olfactory ensheathing cells tightly regulate estradiol levels in the vicinity of the OSNs, which may be their novel role. These cells also abundantly express Star gene, denoting their capacity for cholesterol synthesis. Although all olfactory bulb cells lack Hsd17b6 enzyme to inactivate estradiol and androgens, some cells such as astrocytes, oligodendrocytes, and olfactory ensheathing cells have the capacity to inactivate estradiol using other enzymes. These cells can also produce DHEA, a precursor of androgens. High expression of Hsd17b12 enzyme which converts estrone to active estradiol in the OPC, astrocytes, and oligodendrocytes indicate the importance of estrogens in these OB cells, with further implication for potential estrogen mediation of olfactory processing.

Aging increases the expression of progesterone and androgen receptors.

No major sex differences in genes encoding steroidogenic enzymes, sex steroid receptors, and signaling molecules between female and male mice were found in the olfactory epithelium, implying that sex differences in the olfactory sensitivity are a result of the fluctuation of circulating sex steroids rather than of a differential molecular signature of steroidogenic genes.

A sexually dimorphic lateral nasal gland secretes high levels of testosterone and secretoglobins in males. In particular, Scbg1b27 gene expression is much higher in males than females, and sex-combined housing results in a reduction in the difference.

Aromatase activity and consequent modulation of the androgen-to-estrogen ratio within the AOB are involved in the regulation of sensory representations at the olfactory bulb level, and therefore, they may give rise to sex differences. The activity of Grueneberg neurons which project their axons to the necklace glomeruli may be modulated by aromatase. The identity and confirmation of the putative aromatase-positive glomeruli would provide insight into their regulation by sex-steroid hormones.

Circulating sex hormones have positive effects on OSN development, maturation, and general morphology. However, their effects on odor-induced glomerular activation appear to be the opposite. Circulating estradiol increases, while testosterone decreases glomerular activity, indicating sexually dimorphic sensory circuitry in the olfactory bulb. Furthermore, available data suggest in situ steroidogenesis in the MOB, hence indicating potential modulation of olfactory signal processing, though the details of this remain to be discovered.

The existence of sexually dimorphic circuits at the level of the AOB and medial amygdala has been demonstrated in several recent studies (Unger et al. 2015; Billing et al. 2020; Inbar et al. 2022). As both circulating sex steroids and neurosteroids contribute to sex differences in social interactions and sex-typical behaviors, the significance of aromatase cannot be overestimated. However, low expression of aromatase in the OSNs argues against aromatase role at the peripheral level. In general, aromatase expression is higher in male than in female brains (Wu et al. 2009). As aromatase ultimately produces estrogens from androgens, it is possible that estrogens produced locally in the aromatase-positive periglomerular cells of the OB, which exert inhibitory effects on the odor-activated glomerular activity, result in a lower number of activated glomeruli seen in males (Kass et al. 2017). As aromatase-positive neuronal circuits and the social behavior network process chemosensory cues—which results in the specific and sometimes sexually dimorphic behavioral response—it would be reasonable for them to also modulate olfactory processing in the olfactory bulb, and therefore, they could also contribute to the overall behavioral response. Future research is necessary to test this hypothesis.

## Abbreviations

5α DHP: 5 Alpha dhydroprogesterone
A4: Androstenedione
A5: Androstenediol
ADT: Androsterone
Akr1c18: Alpha ketoreductase
ALLOP: Allopregnanolone
AOB: Accessory olfactory bulb
Ar: Androgen receptor
Cyp11a1: Cytochrome P450, family 11, subfamily a, polypeptide 1
Cyp17a1: Cytochrome P450, family 17, subfamily a, polypeptide 1
Cyp19a1: Cytochrome P450, family 19, subfamily a, polypeptide 1
Cyp2a5: Cytochrome P450, family 2, subfamily a, polypeptide 5
Cyp2g1: Cytochrome P450, family 2, subfamily g, polypeptide 1
DHEA: Dehydroepiandrosterone
DHEAS: Dehydroepiandrosterone sulfate
DHT: Dihydrotestosterone
E1: Estrone
E1S: Estrone sulfate
E2: Estradiol
Epi-ADT: Epi-androsterone
Esr: Estrogen receptor
GABA: Gamma aminobutyric acid
GDX: Gonadectomy
GG: Grueneberg ganglion
Gper 1: G protein-coupled estrogen receptor 1
GSEA: Gene set enrichment analysis
Hsd17b: Hydroxysteroid dehydrogenase
Hsd3a: 3α Hydroxysteroid dehydrogenase
INP: Immature neuronal precursor cells
LNG: Lateral nasal gland
MOB: Main olfactory bulb
MS4A: 4-Pass transmembrane protein receptor
OB: Olfactory bulb
OE: Olfactory epithelium
OPC: Oligodendrocytes precursor cells
OSN: Olfactory sensory neuron
P4: Progesterone
Pagr: Progestin and adipoQ receptor family
PAPS: 3’-Phospho-5’-adenylyl sulfate
Pgr: Nuclear progestin receptor
Pgrmc1: Progesterone receptor membrane component 1
Shbgs: Steroid hormone-binding globulins
Soat1: Sterol O acyltransferase 1
SOM: Septal organ of Masera
Srd5a: 5 Alpha reductase
Srebf: Sterol regulatory element-binding transcription factor
STSs: Sulfatases
Sults: Sulfotransferases
T: Testosterone
TRPM8: Transient receptor potential cation channel subfamily M, member 8
Ugt2a1: UDP glucuronosyltransferase family 2 member a1
V-SVZ: Ventricular-subventricular zone
VNO: Vomeronasal organ
OM: Olfactory mucosa
